# Perceived impact of mass media campaign messages on smoking and mental health: an online survey

**DOI:** 10.1186/s12889-026-27662-0

**Published:** 2026-05-12

**Authors:** Stephanie Fincham-Campbell, Leonie Brose, Charity Aienobe-Asekharen, Hannah Walsh, Ann McNeill, Debbie Robson

**Affiliations:** 1 Addictions, Institute of Psychiatry, Psychology and Neuroscience (IoPPN), King’s College London, 4 Windsor Walk, London, SE5 8BB UK; 2 National Centre for Sport and Exercise Medicine, Loughborough University, Towers Way, Loughborough, Leicestershire, LE11 3TU UK

**Keywords:** Mass media, health promotion, smoking cessation, mental health

## Abstract

**Background:**

A common reason for smoking is perceived mental health benefits but systematic reviews have shown that stopping smoking is associated with mental health benefits such as a reduction in symptoms of anxiety and depression. This message is rarely included in mass media campaigns (MMC) for stopping smoking which largely focus on physical health. We developed animatics (early versions of videos) about the relationship between smoking cessation and mental health and evaluated several perceived impact outcomes.

**Methods:**

Two short MMC animatics (one informational, one testimonial) were co-developed using a multi-stage process, with experts by experience and profession. In an online survey, n=1504 participants watched these two alongside an existing animatic (stress-cycle) in a randomised order. All participants currently or previously smoked or currently vaped; those with mental health conditions were oversampled (73% ≥1 mental health diagnosis, 70% past-month psychological distress). Primary outcome: perceived effectiveness (six items rated from strongly disagree (1) to strongly agree (5)). Secondary outcomes included preferred animatic, relatability, and feeling a) more motivated to stop smoking, b) encouraged to seek professional support, c) more positive about the mental health benefits of stopping smoking.

**Results:**

All three animatics scored > 3.5 on perceived effectiveness with no difference by mental health measures. The informational animatic scored higher on some outcomes, with all effects being small or very small. There were no significant effects for ever diagnosis for any secondary outcome. People with psychological distress rated the informational and stress-cycle animatics higher than those with no or low distress. Each animatic was a favourite for about one-third of respondents, regardless of mental health status.

**Conclusions:**

Smoking cessation campaign messages highlighting mental health benefits were well-received across people with and without mental health conditions, suggesting potential to reduce smoking prevalence.

**Supplementary Information:**

The online version contains supplementary material available at 10.1186/s12889-026-27662-0.

## Background

Globally, smoking is a leading risk factor for premature mortality and morbidity [1, 2]. Half of the people who smoke will die prematurely if they continue to smoke [3]. In 2020, it was estimated that worldwide, 1.18 billion people regularly smoked tobacco [4] and population growth means the absolute number continues to rise [1]. Within this global framework, there are mental health inequalities, for example, in England, smoking prevalence in 2024 was 25.1% among those with a long-standing mental health condition compared to 11.6% in the population overall [5]. Other countries, including the United States [6–8], and Australia [9], report similar disparities. Additionally, while smoking prevalence has declined over time, these reductions have been markedly smaller among people with mental health conditions [10–12]. People with severe mental illness have much shorter life expectancies, with smoking accounting for up to two-thirds of this gap [13–16] .

A common reason cited for smoking is perceived mental health benefits, including alleviating emotional problems and feelings of depression and anxiety, stabilising mood, relaxing, and relieving stress [17]. However, systematic reviews show that stopping smoking is associated with improvements in mental health [17, 18]. Additionally, a nationally representative household survey found evidence that improvements in mental health may increase over time [19] which may be more pronounced among those with an ever diagnosis of a mental health condition. The misconception that smoking benefits mental health can at least partly be explained by the relief smoking provides from nicotine withdrawal [19–21]. There is some evidence that believing smoking helps people to cope may be associated with lower cessation rates [22].

Given the benefits of stopping smoking and misconceptions around mental health, it is important to consider how best to trigger and support cessation among people with mental health conditions, including through public health strategies. Tobacco control mass media campaigns (MMC) are effective at increasing quit attempts and cessation [23, 24]. However, little is known about their effectiveness among people with mental health conditions. A systematic review of the effect of tobacco control MMC on smoking-related behaviour among people with mental health conditions identified only eight eligible studies [25]. The findings suggested that while general tobacco control campaigns had limited impact, a campaign with a narrator identifying as having a mental health condition was associated with greater intention to quit and making a quit attempt [26]. Further support for this finding comes from an experimental study in which people with mental health conditions were more motivated to stop smoking when they saw a message focused on the mental health benefits of stopping smoking compared to a message focused on physical health benefits [27].

Our study aimed to add to these promising findings by developing two MMC animatics (early versions of videos that combine storyboard images with basic timing, sound, and voiceover) and evaluating their perceived impact, together with an existing UK animatic [28], among people with and without mental illness in an online survey. We aimed to assess overall perceptions of these animatics including an overall measure of perceived effectiveness, and perceptions of impact on motivation to stop smoking and the mental health benefits of stopping. We also assessed differences in perceptions between animatics and by mental health status.

## Methods

### Development of animatics

The two animatics were developed through a multi-stage process and informed by the previous systematic review [25]. They were co-developed with experts by experience (i.e. people with lived experience of mental health conditions), given the need to balance the effectiveness of messaging against the risk of stigmatising [29] and informed by professionals with expertise in smoking and mental health. First, we conducted an online survey (unpublished) with 1,414 people with self-reported mental health conditions, and 978 without, to explore how engaging a varied selection of existing smoking-related MMC were, including two campaigns with mental health messages [26, 30]. We also consulted research on the development of health warning labels [29, 31, 32]. We then discussed our findings with a bespoke experts by experience group. Based on this information, we drafted eight candidate messages that were either informational or testimonial in style, which the experts by experience group refined further. A separate lived experience group [33] and 24 professionals working in smoking cessation, mental health and/or MMC then rated the messages on accuracy, clarity, attention-grabbing, and relatability/resonance. Two final messages were selected based on those scores and further developed into storyboards and animatics by a social marketing company (DIVA Creative, Sheffield, UK) with multiple rounds of revision informed our experts by experience group.

### Evaluation of messages

An Open Science Framework (OSF) pre-registration (10.17605/OSF.IO/BCPN3) provides additional detail.

### Design and recruitment

Data were collected in August to September 2024 using an online cross-sectional survey. Participants were recruited using convenience sampling via Prolific Academic, a crowdsourcing platform with its own global pool of research participants who are verified using ID checks and vetted using attention, comprehension and honesty checks [34].

Prolific has pre-set screener questions which were used to send the survey to people aged 18 and over in the UK who currently used tobacco products and/or e-cigarettes, or had previously used tobacco products, and to oversample participants with a history of mental illness, aiming for two thirds of the sample. Prolific Academic reimbursed participants who completed the survey at £9.50 per hour.

### Materials and procedure

**Fig. 1:**
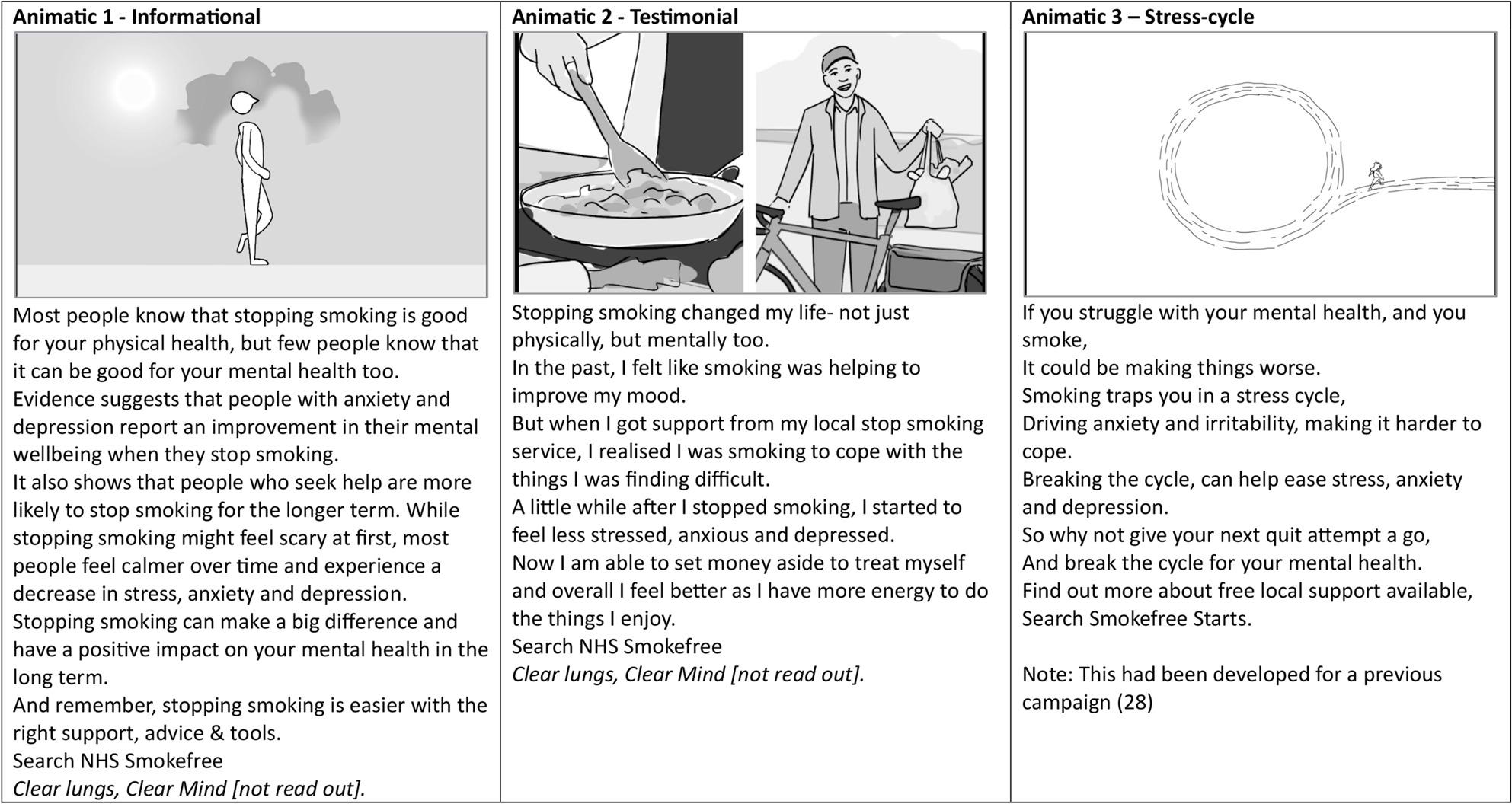
Screenshots and transcripts for the three animatics

Three animatics were included; 1 and 2 were developed as detailed above, and 3 was from an existing regional campaign, also developed by DIVA [28], to ensure comparability. Screenshots and transcripts are provided in Fig. 1.

1. Informational: The voice-over disseminated information about the links between stopping smoking and improvements in mental health. The imagery showed schematic lungs, a brain, and a body clearing over time, and a walking person transitioning from a slumped to an upright, energetic position.

2. Testimonial: A testimonial by a male person who had stopped smoking. He talked about how stopping smoking had reduced feelings of stress, anxiety and depression and improved finances to do things he enjoyed with imagery showing the narrator cycling and cooking.

3. Stress-cycle: The voice-over described how smoking can trap people in a cycle of stress, and breaking the cycle can help ease stress, anxiety and depression. It showed people walking in a circle with their heads covered in smoke, which dissipated, and they were then able to walk out of the circle.

All animatics included prompts to find out more about local smoking cessation support. Participants were shown all three animatics in randomised order and completed the outcome measures after each one.

Measures.

The questionnaire and outcome measures were co-developed with the experts by experience group for this study and is available on OSF (10.17605/OSF.IO/BCPN3 and Supplementary File 1).

### Primary outcome – perceived effectiveness

Perceived effectiveness was measured using six items: worth remembering, attention-grabbing, powerful, informative, meaningful and convincingness, each rated on a five-point scale [Strongly disagree [1], Disagree [2], Neither agree nor disagree [3], Agree [4], Strongly agree [5] ]. The average across the six items yielded a total perceived effectiveness score. This score has construct validity for smoking cessation campaign advertisements [35] and predicts subsequent quit attempts [36]. In the present survey, Cronbach’s alpha was above 0.9, indicating high internal consistency.

### Secondary outcomes

These included that the respondent felt (a) they could relate to the animatic, (b) they were more motivated to stop smoking, (c) encouraged to seek professional support to stop smoking, and (d) more positive about the mental health benefits of stopping smoking. Each was rated on the same five-point scale as the primary outcome. Respondents were also asked to select their favourite animatic.

### Mental health

Ever diagnosis of mental health condition: From a list of conditions, participants selected those that a doctor or healthcare professional had ever told them they had. Responses were recoded into none of these, one, or two or more of the conditions [37, 38].

Psychological distress: Measured by the six-item version of the Kessler Psychological Distress Scale (K6). Scores between 0 and 4 recoded as no/low distress, 5–12 as moderate distress and 13–24 as serious distress experienced in the past month [39–41].

### Further characteristics and attention check

Socio-demographics included gender (Male, Female, Non-binary, Prefer to self-describe, collapsed into male versus all others for regressions), ethnic group (Asian/Asian British, Black/African/Caribbean/Black British, Mixed/Multiple, White, Other, for regressions collapsed into racialised minorities and white), country (England, Northern Ireland, Scotland, Wales), level of education (High, defined as university degree or equivalent, Bachelor’s degree or equivalent, Master’s degree, PhD or equivalent; Medium, defined as A-level or equivalent, and Low, defined as GCSE/O-level/CSE, vocational qualifications, other, no formal qualifications) and current employment status (Employed/Self-employed, Unemployed, Retired/Student/Other).

Smoking and vaping status was categorised into Smoking and vaping, Exclusive smoking, Exclusive vaping, or Neither (past smoking). Urges to smoke [42] were categorised as None, Slight, Moderate, or Strong, and Motivation to stop smoking [43, 44] into Motivated to stop within 3 months or Not motivated to stop within 3 months.

### Two attention checks were included; participants failing both would have been excluded

#### Sample

The survey was started by 1,614 participants and completed by 1,519. Fifteen were excluded for missing data (‘prefer not to say’) on socio-demographics, smoking or vaping measures, leaving *n* = 1,504 for analyses including ever diagnosis of a mental health condition. This sample size provided 95% power (α = 0.05) to detect a small effect [45]. Analysis using psychological distress (*n* = 1,451) excluded ‘don’t know’ or ‘prefer not to say’ responses to any of the six items.

#### Analysis

Analyses used SPSS v29. Percentages and frequencies were used to describe the sample and percentages, means, and standard deviations to describe outcomes.

General Linear Models were used to compare total perceived effectiveness, individual effectiveness items and secondary outcomes (except favourite animatic) across the three animatics. These were then repeated with animatics as the within-subjects factor and mental health (separate models for ever diagnosis and psychological distress) as the between-subjects factor. Alpha was set at 0.05, and effect size partial eta squared calculated [46]. A one-sample proportions test was used to test whether any of the three videos emerged as an overall favourite, and chi-square tests were used to assess differences in preference by ever diagnosis and psychological distress.

Some changes to the pre-registered analysis plan were made: To reduce survey burden and measure overlap, we did not ask about believability; we coded gender as male (instead of female) versus all others; and we used General Linear Models instead of linear and logistic regressions for consistency across outcomes. We stopped recruitment with sufficient power at about 1500 instead of 1800.

## Results

### Sample description

Participants were 43% male, two-thirds were aged 25–44 (65%), and most were white (85%). Most participants resided in England (86%) and were employed (80%); just over half had a high level of education (55%). Most respondents had ever had a diagnosis of at least one mental health condition (73%), and experienced moderate/serious psychological distress in the past month (70%, Table 1). Many participants currently smoked tobacco (61%), and most reported vaping (85%). About half smoked and vaped (53%), 32% exclusively vaped (of whom 98% had previously smoked), and some exclusively smoked (8%). Few who smoked were motivated to stop within the next three months (9%). Among people who smoked or had recently stopped smoking, a third reported strong urges to smoke (33%, Table 1).

**Table 1:** Participants’ socio-demographic, mental health, smoking and vaping characteristics (*N* = 1504)

Characteristic	Group	% (*n*)
Gender	Male	42.6 (640)
	Female	56.3 (847)
	Other	1.1 (17)
Age	18–24	10.0 (151)
	25–34	38.4 (578)
	35–44	26.1 (392)
	45–54	15.6 (235)
	55 and over	9.8 (148)
Ethnicity	Asian/Asian British	5.7 (85)
	Black/African/Caribbean/Black British	4.3 (65)
	Mixed/Multiple	4.1 (61)
	White	84.8 (1276)
	Other	1.1 (17)
Country	England	86.0 (1294)
	Northern Ireland	2.3 (34)
	Scotland	7.0 (106)
	Wales	4.6 (70)
Level of education	High	54.9 (826)
	Medium	26.5 (399)
	Low	18.6 (279)
Employment status	Employed/Self-employed	80.2 (1206)
	Unemployed	11.2 (169)
	Retired/Student/Other	8.6 (129)
Current smoking/vaping	Smoking and vaping	53.1 (798)
	Exclusive smoking	7.5 (113)
	Exclusive vaping	32.0 (482)
	Neither (past smoking)	7.4 (111)
Motivation to stop smoking ^a^	Within 3 months	8.6 (77)
	Not within 3 months	91.4 (823)
Urges to smoke ^b^	None	24.2 (360)
	Slight	15.2 (226)
	Moderate	27.4 (409)
	Strong	33.2 (495)
Ever mental health diagnosis	None	27.0 (406)
	One	17.0 (255)
	Two or more	56.1 (843)
Psychological distress ^c^	No/low	29.8 (432)
	Moderate	44.9 (652)
	Serious	25.3 (367)

^a^: Only asked of people who currently smoked (*n* = 911, including 11 responding ‘don’t know’ who were excluded)

^b^: Only asked of people who currently smoke or stopped smoking within the last year (*n* = 1491, including 1 ‘don’t know’ who was excluded)

^c^: *n* = 1,451 due to exclusion of people who preferred not to respond or were unsure (*n* = 53)

### Primary outcome: perceived effectiveness

All three animatics scored above 3.5. The informational animatic [M (SD) = 3.70 (0.78)] scored significantly higher than the testimonial animatic [M (SD) = 3.57 (0.83); mean difference = 0.13, 95% CI: 0.09 to 0.18, *p* < .001] and the stress cycle animatic [M (SD) = 3.58 (0.83); mean difference = 0.12, 95% CI: 0.08 to 0.17, *p* < .001; Table 2]; however mean differences and effect sizes were small at best. No significant differences for perceived effectiveness were found by ever diagnosis (F(2,1501) = 0.99, *p* = .37, η²= 0.001, Table 3) or psychological distress (F(2,1448) = 0.33, *p* = .72, η²<0.001, Table 4) for the overall measure. There was a small but significant effect of ever diagnosis on the item ‘informative’ (Table 3); respondents with two or more diagnoses rated the testimonial animatic lower than those with no diagnoses. There were no other significant effects of diagnosis or psychological distress on any of the individual items (Table 4).

**Table 2:** Outcome measures across the three videos, *n* = 1504

	Informational, Mean (SD)	Testimonial, Mean (SD)	Stress-cycle, Mean (SD)	Comparison ^2^
Perceived effectiveness ^1^	3.70 (0.78)	3.57 (0.83)	3.58 (0.83)	F(2,3006) = 26.46, *p*<.001, partial η²=0.017
Individual perceived effectiveness items				
Worth remembering	3.76 (0.89)	3.64 (0.95)	3.66 (0.91)	F(2,3006) = 13.53, *p*<.001, partial η²=0.009
Attention-grabbing	3.53 (1.03)	3.43 (1.10)	3.51 (1.07)	F(2,3006) = 7.26, *p*<.001, partial η²=0.005
Powerful	3.38 (1.03)	3.32 (1.08)	3.31 (1.05)	F(2,3006) = 4.20, *p*=.015, partial η²=0.003
Informative	4.10 (0.79)	3.70 (0.94)	3.83 (0.90)	F(2,3006) = 143.74, *p*<.001, partial η²=0.087
Meaningful	3.82 (0.89)	3.82 (0.90)	3.73 (0.93)	F(2,3006) = 9.82, *p*<.001, partial η²=0.006
Convincing	3.60 (0.99)	3.51 (1.04)	3.44 (1.05)	F(2,3006) = 19.02, *p*<.001, partial η²=0.012
Relatable	3.62 (1.01)	3.61 (1.07)	3.48 (1.08)	F(2,3006) = 15.21, *p*<.001, partial η²=0.010
More motivated to stop	3.28 (1.07)	3.20 (1.09)	3.15 (1.09)	F(2,3006) = 11.54, *p*<.001, partial η²=0.008
Encouraged to seek support	3.10 (1.09)	2.94 (1.07)	2.96 (1.10)	F(2,3006) = 24.41, *p*<.001, partial η²=0.016
More positive about mental health benefits	3.80 (0.97)	3.67 (1.03)	3.63 (1.05)	F(2,3006) = 25.70, *p*<.001, partial η²=0.017

^1^Primary outcome, average of the six individual perceived effectiveness items

^2^Effect size partial eta squared benchmarks: 0.01 (small), 0.06 (medium), and 0.14 (large) [46]

**Table 3:** Outcome measures across the three animatics by mental health diagnosis, *n* = 1504

	Informational, Mean (SD)			Testimonial, Mean (SD)			Stress-cycle, Mean (SD)			Main effect
	Ever mental health diagnosis			Ever mental health diagnosis			Ever mental health diagnosis			
	0	1	≥ 2	0	1	≥ 2	0	1	≥ 2	
Perceived effectiveness	3.78 (0.76)	3.69 (0.81)	3.67 (0.78)	3.60 (0.85)	3.58 (0.82)	3.55 (0.85)	3.57 (0.85)	3.54 (0.86)	3.59 (0.82)	F(2,1501) = 0.99, *p*=.372, partial η²=0.001
Worth remembering	3.81 (0.87)	3.75 (0.92)	3.74 (0.90)	3.62 (0.94)	3.66 (0.96)	3.65 (0.95)	3.64 (0.96)	3.61 (0.93)	3.68 (0.89)	F(2,1501) = 0.04, *p*=.965, partial η²<0.001
Attention-grabbing	3.65 (0.99)	3.50 (1.02)	3.50 (1.04)	3.46 (1.10)	3.42 (1.11)	3.41 (1.10)	3.50 (1.07)	3.44 (1.11)	3.55 (1.06)	F(2,1501) = 0.94, *p*=.390, partial η²=0.001
Powerful	3.49 (1.01)	3.37 (1.03)	3.33 (1.02)	3.36 (1.09)	3.32 (1.07)	3.29 (1.08)	3.24 (1.08)	3.29 (1.06)	3.34 (1.03)	F(2,1501) = 0.40, *p*=.673, partial η²=0.001
Informative	4.16 (0.73)	4.09 (0.81)	4.08 (0.81)	3.80 (0.92)	3.67 (0.91)	3.66 (0.96)	3.91 (0.87)	3.81 (0.96)	3.80 (0.90)	F(2,1501) = 3.63, *p*=.027, partial η²=0.005
Meaningful	3.90 (0.86)	3.88 (0.90)	3.77 (0.90)	3.83 (0.91)	3.86 (0.84)	3.80 (0.92)	3.76 (0.93)	3.70 (0.93)	3.73 (0.93)	F(2,1501) = 1.11, *p*=.330, partial η²=0.001
Convincing	3.69 (1.00)	3.55 (0.99)	3.59 (0.98)	3.51 (1.06)	3.55 (1.00)	3.50 (1.05)	3.51 (1.05)	3.38 (1.09)	3.42 (1.04)	F(2,1501) = 1.01, *p*=.348, partial η²=0.001
Relatable	3.55 (0.99)	3.59 (0.99)	3.65 (1.02)	3.56 (1.08)	3.64 (1.03)	3.62 (1.08)	3.37 (1.09)	3.43 (1.09)	3.55 (1.08)	F(2,1501) = 2.36, *p*=.095, partial η²=0.003
More motivated to stop	3.34 (1.05)	3.27 (1.08)	3.25 (1.09)	3.24 (1.07)	3.21 (1.09)	3.17 (1.10)	3.13 (1.11)	3.18 (1.08)	3.15 (1.09)	F(2,1501) = 0.41, *p*=.666, partial η²=0.001
Encouraged to seek support	3.10 (1.09)	3.08 (1.09)	3.11 (1.09)	2.95 (1.09)	2.87 (1.04)	2.96 (1.06)	2.92 (1.13)	2.93 (1.07)	2.99 (1.10)	F(2,1501) = 0.38, *p*=.684, partial η²=0.001
More positive about mental health benefits	3.84 (0.95)	3.80 (0.94)	3.78 (0.99)	3.66 (1.03)	3.71 (0.94)	3.66 (1.05)	3.63 (1.06)	3.59 (1.03)	3.63 (1.05)	F(2,1501) = 0.07, *p*=.930, partial η²<0.001

**Table 4:** Outcome measures across the three animatics by psychological distress, *n* = 1451

	Psychological distress			Psychological distress			Psychological distress			
	No/low	Moderate	Serious	No/low	Moderate	Serious	No/low	Moderate	Serious	
Perceived effectiveness	3.76 (0.77)	3.68 (0.78)	3.70 (0.78)	3.60 (0.83)	3.56 (0.81)	3.57 (0.88)	3.57 (0.83)	3.58 (0.84)	3.61 (0.80)	F(2,1448) = 0.33, *p*=.718, partial η²<0.001
Worth remembering	3.80 (0.83)	3.73 (0.91)	3.78 (0.93)	3.65 (0.92)	3.63 (0.92)	3.68 (1.00)	3.63 (0.92)	3.64 (0.91)	3.74 (0.88)	F(2,1448) = 0.96, *p*=.384, partial η²=0.001
Attention-grabbing	3.59 (1.00)	3.49 (1.04)	3.56 (1.02)	3.44 (1.11)	3.44 (1.07)	3.41 (1.12)	3.48 (1.04)	3.54 (1.08)	3.54 (1.07)	F(2,1448) = 0.06, *p*=.942, partial η²<0.001
Powerful	3.44 (1.00)	3.35 (1.01)	3.37 (1.05)	3.33 (1.09)	3.30 (1.05)	3.35 (1.10)	3.25 (1.05)	3.32 (1.04)	3.35 (1.05)	F(2,1448) = 0.18, *p*=.839, partial η²<0.001
Informative	4.15 (0.72)	4.09 (0.80)	4.12 (0.81)	3.77 (0.95)	3.66 (0.90)	3.69 (0.98)	3.87 (0.86)	3.82 (0.92)	3.85 (0.88)	F(2,1448) = 1.53, *p*=.271, partial η²=0.002
Meaningful	3.87 (0.88)	3.83 (0.87)	3.81 (0.89)	3.88 (0.85)	3.81 (0.87)	3.81 (0.95)	3.75 (0.91)	3.71 (0.95)	3.77 (0.91)	F(2,1448) = 0.63, *p*=.535, partial η²=0.001
Convincing	3.68 (1.00)	3.60 (0.95)	3.57 (1.01)	3.53 (0.99)	3.52 (1.05)	3.49 (1.05)	3.45 (1.06)	3.46 (1.06)	3.42 (1.02)	F(2,1448) = 0.465, *p*=.628, partial η²=0.001
Relatable	3.53 (1.01)	3.63 (0.99)	3.71 (0.99)	3.52 (1.07)	3.65 (1.02)	3.68 (1.10)	3.31 (1.11)	3.51 (1.07)	3.66 (1.03)	F(2,1448) = 8.03, *p*<.001, partial η²=0.011
More motivated to stop	3.23 (1.01)	3.30 (1.08)	3.31 (1.10)	3.13 (1.06)	3.24 (1.07)	3.23 (1.11)	3.04 (1.07)	3.20 (1.09)	3.23 (1.10)	F(2,1448) = 2.54, *p*=.079, partial η²=0.003
Encouraged to seek support	3.01 (1.07)	3.11 (1.05)	3.18 (1.15)	2.86 (1.07)	2.95 (1.03)	3.02 (1.12)	2.85 (1.11)	3.00 (1.08)	3.01 (1.12)	F(2,1448) = 3.38, *p*=.034, partial η²=0.001
More positive about mental health benefits	3.81 (0.94)	3.82 (0.96)	3.78 (1.00)	3.62 (1.03)	3.70 (1.00)	3.70 (1.03)	3.57 (1.02)	3.66 (1.06)	3.66 (1.05)	F(2,1448) = 0.72, *p*=.485, partial η²=0.001

^1^Effect size partial eta squared benchmarks: 0.01 (small), 0.06 (medium), and 0.14 (large) [46]

### Secondary outcomes

All three animatics scored above the midpoint (neither agree nor disagree) for relatability, feeling more motivated to stop smoking, and feeling more positive about the mental health benefits of stopping. Scores were just below for feeling more encouraged to seek support to stop smoking for the testimonial and stress-cycle animatics (Table 2). The informational animatic was rated higher than the stress-cycle animatic on relatability and higher than the other two animatics on feeling more motivated to stop smoking, more encouraged to seek support, and feeling more positive about the mental health benefits of stopping; however, even the largest difference represented a small effect. As some secondary outcomes were less applicable to people not currently smoking, we repeated analyses with the sample restricted to those who smoked (*n* = 911). None of the conclusions changed.

There were no significant effects for ever diagnosis for any secondary outcome (Table 3). For psychological distress, a significant effect was found for relatability (Table 4); those with serious distress rated the informational and the stress-cycle animatic higher than those with no or low distress, and those with moderate distress rated the stress-cycle animatic higher than those with no/low distress. For feeling encouraged to seek support, while there was an overall effect, none of the pairwise comparisons reached significance.

Each animatic was the favourite for similar proportions (informational: 31.1%, 95% CI: 28.8 to 33.5, testimonial: 34.2%, 95% CI: 31.9 to 36.7, stress-cycle: 34.6%, 95% CI: 32.3 to 37.1), with none of these proportions significantly different from 33% and no significant variation by ever diagnosis (χ^2^ = 2.78, *p* = .60) or psychological distress (χ^2^ = 3.704, *p* = .45).

## Discussion

Two animatics (informational and testimonial) challenging the widely held view that smoking benefits mental health were co-developed with experts by experience and professional stakeholder input, and tested alongside a third animatic (stress-cycle). All were perceived as effective and differences in perceived effectiveness were small. Approximately equal proportions of participants rated each animatic as their favourite. The informational animatic scored higher than the other two on perceived effectiveness and four secondary outcomes; however, all differences were small, and this may partly be due to the slightly longer narrative in this animatic. Importantly, for the primary outcome and most secondary outcome measures, there were no significant differences when considering mental health (using two indicators: ‘ever diagnosis’ and ‘psychological distress’), suggesting animatics were broadly acceptable regardless of mental health status.

The seemingly intractable differences in smoking among people with and without mental health conditions require more focus to ensure that smokefree targets include those who experience poor mental health. The two co-developed animatics gave similar messages that stopping smoking is associated with improved mental health, although one had a third person narration and the other a first person narration. A third animatic, developed independently as a regional campaign, focused on the myth that smoking relieves stress. The positive ratings for the messages included across the three animatics are encouraging, given the relationship between perceived effectiveness and cessation behaviours.

There were only small differences in some outcomes across animatics and mental health conditions, indicating that the messages were well received across the spectrum of mental health, suggesting that messages on mental health can be effective for all people who smoke. This is in line with and builds on research by Steinberg (2024) [27], which found no significant effects of mental health conditions on perceived effectiveness, showing that messages conveying the mental health benefits of stopping smoking are well received by people who smoke with mental health conditions and increase motivation to stop smoking. Additionally, we found that the messages could increase interest in seeking support, which is important as this will maximise chances of successful stopping, particularly given greater dependence on smoking among people with mental health conditions [10, 11, 38]. We also found that the animatics were relatable and that participants were more positive about the mental health benefits of stopping smoking after viewing them. Encouragingly, participants with higher distress rated one or more animatics higher than those without distress on relatability and encouragement to seek support.

In England, the importance of MMCs for increasing smoking cessation was recently acknowledged, and funding increases were announced for new national campaigns [47]. This presents an opportunity to include messages on the mental health benefits of smoking cessation, given widespread misperceptions about smoking improving mental health, and that previous campaigns had focused largely on the physical health consequences of smoking. Since approximately a third of the survey sample rated each animatic as their favourite, having several MMCs focusing on mental health might broaden appeal and reach. Additional research may identify ways to increase perceived effectiveness and appeal further as all outcome measures had potential for higher ratings.

### Strengths and limitations

We successfully oversampled participants with experience of mental health problems who are often underrepresented in population surveys and achieved good representation of socio-demographics and smoking characteristics (e.g. [38, 48, 49]); however, the online sample may limit generalisability of study findings. All measures, including mental health status, were self-reported and may have been subject to recall errors and misreporting. While we did not assess level of attention paid to the animatics which may limit validity of the findings, the survey would not move on until the animatic had finished, and we included two general attention checks. This survey tested self-reported reactions to animatics, which may not translate into behavioural outcomes following observation of MMCs in the real world. We did not include a neutral or inert animatic, so we cannot assess whether the observed effects are specific to the content of the smoking and mental health messages. Including a neutral comparator would have helped to isolate the active components of the messages. Our measure of perceived effectiveness had construct validity and had been shown to predict quit attempts, however, it does not test actual behaviour. Nevertheless, the findings here, together with Steinberg et al. [27] indicate that campaigns should communicate the mental health benefits of smoking cessation as they were well received by participants and have the potential for wide reach and impact.

## Conclusions

The burden of tobacco smoking is disproportionately carried by people with mental health conditions. We developed and tested smoking cessation mass media campaign animatics conveying the associations between stopping smoking and improvements in mental health, challenging assumptions that smoking improves mental health. Participants responded positively across a range of outcome measures including perceived effectiveness, with few differences across those with and without mental health conditions. The animatics were also reported to increase motivation to quit and help seeking. Messages focusing on smoking and mental health would therefore have the potential for wide impact across a population with high smoking prevalence and experiencing considerable smoking-related health inequalities.

## Supplementary Information

Supplementary Material 1.

## Data Availability

The datasets used and analysed during the current study are available from the corresponding author on reasonable request.
