# Supplementary material for: Perceived impact of mass media campaign messages on smoking and mental health: an online survey
Source: BMC Public Health. 2026 May 12;26:2040. doi: 10.1186/s12889-026-27662-0 (PMC13335273; doi:10.1186/s12889-026-27662-0)
Supplement: Supplementary file 1 — Supplementary Material 1. [file 12889_2026_27662_MOESM1_ESM.docx]

**Mass Media Campaign Survey**

Ethical review reference number: HR-23/24-42746

Version number: 1.4, 20/08/2024

**Aim**: To find out what people think about different messages about smoking and mental health which could be used in mass media campaigns (such as television, radio, social media and the internet).

**What will you see in the Survey**: 3 short video clips which could be used for a mass media campaign. These were made with people with lived experience of smoking and mental illness and other professionals. The videos are still in the early stages of development and will be improved before they are released more widely. You will be asked to give feedback on the video clips.

**What questions will you see**: You will be asked some questions to enable you to decide if you want to participate or not, and then you will be asked questions about your smoking and vaping. You will also be asked questions about your background and your mental health status. The survey will take you approximately 20 minutes to complete.

**Consent**

Please download the information sheet document and check the relevant option.

*Forced response for each. If the response for ≥1 statement is No, participants will be directed to the end of the survey page.*

- I confirm that I have read and understood the [information sheet](https://emckclac-my.sharepoint.com/:w:/g/personal/k2371738_kcl_ac_uk/EcHppCJxK9FOp42hDq6xLdcBFqZocTyRhlbAaJExPFaAYQ?e=rrMKSR) dated 02/08/2024, V1.3 for the project. I have had the opportunity to consider the information and asked questions which have been answered to my satisfaction.


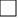
Yes


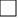
No

- I agree to take part in this survey and understand that I can choose not to participate or withdraw at any time before the results are reviewed, without needing to give a reason.


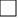
Yes


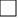
No

- I consent to the processing of my personal information (including information from the pre-screening questions) for the purposes explained to me in the information sheet. I understand that such information will be handled under the terms of UK data protection law, including the UK General Data Protection Regulation (UK GDPR) and the Data Protection Act 2018.


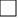
Yes


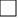
No

- I understand that the information I provide in this survey may be subject to review by individuals from the College for monitoring and audit purposes.


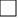
Yes


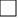
No

- I understand that confidentiality and anonymity will be maintained and protected, and it will not be possible to identify me in any research outputs.


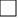
Yes


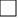
No

- I agree that the research team may use my data for future research (in such cases, as with this project, my personal data would not be identifiable in any report).


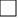
Yes


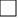
No

***Forced response***

**Please provide your Prolific ID** (the research team will not be able to identify you from your ID number) **_____________**

**Pre-screening validation**

We are asking the following questions to check that your pre-screening responses on Prolific are accurate and up to date. This will ensure that the survey is suitable for you to complete.

**1.** Do you currently live in the United Kingdom (UK)?

- Yes
- No

*(1: directed to Q2. 2: directed to end of survey page).*

**Vaping and Smoking Behaviours**

For the questions in this survey, please note:

- **Vapes/E-cigarettes and vaping:** We **are referring to** nicotine or non-nicotine electronic cigarettes which are also called vapes. We **are not referring to**the use of electronic cigarettes for vaping cannabis or illegal substances
- **Smoke and smoking**: We **are referring to** tobacco cigarettes and other kinds of tobacco that are burned (e.g., pipe, cigar, or shisha). We **are not referring to** electronic or ''heated tobacco'' products (e.g., IQOS) or cannabis smoking.

**2**. This question asks about vapes or e-cigarettes. Could you please tell us which of the following best applies to you now?

- I currently vape or use e-cigarettes every day
- I currently vape or use e-cigarettes, but not every day
- I have tried vaping or using e-cigarette once or a few times
- I stopped vaping or using e-cigarettes completely in the last year
- I stopped vaping or using e-cigarettes completely more than a year ago
- I have never vaped or used e-cigarettes
- Don't know
- Prefer not to say

*(Response Option 7, 8: directed to end of survey page).*

**3.** This question asks about smoking. Which of the following best applies to you?

- I smoke cigarettes (including hand-rolled) every day
- I smoke cigarettes (including hand-rolled), but not every day
- I do not smoke cigarettes at all, but I do smoke tobacco of some kind (e.g. Pipe, cigar or shisha)
- I have stopped smoking completely in the last year
- I stopped smoking completely more than a year ago
- I have never smoked
- Don’t know
- Prefer not to say

*(Response Option 7, 8: Directed to end of survey page; Response Option 6: Directed to the end of the block (i.e. to the attention check question)) If response option 6 is selected in both Q2 & Q3: Directed to end of survey page*

**4.** How strong have your urges been to smoke in the last 24 hours?

- Slight
- Moderate
- Strong
- Very strong
- Extremely strong
- Prefer not to say
- Don't know
- I have not felt the urge to smoke in the last 24 hours

*(Only display Question if response to Q2 & Q3 is vaping and/or smoking; Display Q4 only if response 1, 2, 3, 4, or 5 is selected in Q3)*

**5.** Which of the following describes you?

1. I don’t want to stop smoking

2. I think I should stop smoking but don’t really want to

3. I want to stop smoking but haven’t thought about when

4. I REALLY want to stop smoking but I don’t know when I will

5. I want to stop smoking and hope to soon

6. I REALLY want to stop smoking and intend to in the next 3 months

7. I REALLY want to stop smoking and intend to in the next month

9. Don’t know

*(Display Q5: If response options 1,2, or 3 is selected in Q3)*

**Attention Check**

The following question is to check your level of attention. This will ensure that the data are of good quality.

**A1.** To check your level of attention, please tick **Sometimes**.

- Never
- Rarely
- Sometimes
- Often
- Very often
- Don’t know
- Prefer not to say

**Campaign clips**

You will now watch **3 short video clips** on smoking and mental health, which could be used for Mass Media Campaigns. These clips are still in the early stages of development and the graphics will be improved before released more widely.

After each clip, you will be asked to provide your feedback.

Please use the **caption button (cc)** if you would like to read the text as you watch the clip.

***Embed Video File 1 (Concept 1)***

**6 (A)** The video clip is worth remembering

- Strongly disagree
- Disagree
- Neither agree nor disagree
- Agree
- Strongly agree

**6 (B)** The video clip grabbed my attention

- Strongly disagree
- Disagree
- Neither agree nor disagree
- Agree
- Strongly agree

**6 (C)** The video clip is powerful

- Strongly disagree
- Disagree
- Neither agree nor disagree
- Agree
- Strongly agree

**6 (D)** The video clip is informative

- Strongly disagree
- Disagree
- Neither agree nor disagree
- Agree
- Strongly agree

**6 (E)** The video clip is meaningful

- Strongly disagree
- Disagree
- Neither agree nor disagree
- Agree
- Strongly agree

**6 (F)** The video clip is convincing

- Strongly disagree
- Disagree
- Neither agree nor disagree
- Agree
- Strongly agree

**6 (G)** I can relate to the video clip

- Strongly disagree
- Disagree
- Neither agree nor disagree
- Agree
- Strongly agree

**6 (H)** After watching the video clip, I feel more motivated to stop smoking

- Strongly disagree
- Disagree
- Neither agree nor disagree
- Agree
- Strongly agree

**6 (I)** After watching the video clip, I feel more likely to seek professional support to help me stop smoking

- Strongly disagree
- Disagree
- Neither agree nor disagree
- Agree
- Strongly agree

**6 (J)** After watching the video clip, I feel more positive about the mental health benefits of stopping smoking

- Strongly disagree
- Disagree
- Neither agree nor disagree
- Agree
- Strongly agree

**6 (K)** Do you have any other comments about this clip? Such as what you liked, or disliked (Maximum 1**00 characters**)

________________________________________

***Embed Video File 2 (Concept 2)***

**7 (A)** The video clip is worth remembering

- Strongly disagree
- Disagree
- Neither agree nor disagree
- Agree
- Strongly agree

**7 (B)** The video clip grabbed my attention

- Strongly disagree
- Disagree
- Neither agree nor disagree
- Agree
- Strongly agree

**7 (C)** The video clip is powerful

- Strongly disagree
- Disagree
- Neither agree nor disagree
- Agree
- Strongly agree

**7 (D)** The video clip is informative

- Strongly disagree
- Disagree
- Neither agree nor disagree
- Agree
- Strongly agree

**7 (E)** The video clip is meaningful

- Strongly disagree
- Disagree
- Neither agree nor disagree
- Agree
- Strongly agree

**7 (F)** The video clip is convincing

- Strongly disagree
- Disagree
- Neither agree nor disagree
- Agree
- Strongly agree

**7 (G)** I can relate to the video clip

- Strongly disagree
- Disagree
- Neither agree nor disagree
- Agree
- Strongly agree

**7 (H)** After watching the video clip, I feel more motivated to stop smoking

- Strongly disagree
- Disagree
- Neither agree nor disagree
- Agree

Strongly agree

**7 (I)** After watching the video clip, I feel more likely to seek professional support to help me stop smoking

- Strongly disagree
- Disagree
- Neither agree nor disagree
- Agree
- Strongly agree

**7 (J)** After watching the video clip, I feel more positive about the mental health benefits of stopping smoking

- Strongly disagree
- Disagree
- Neither agree nor disagree
- Agree
- Strongly agree

**7 (K)** Do you have any other comments about this clip? Such as what you liked, or disliked (Maximum **100 Characters**)

________________________________________

**E*mbed Video File 3 (Smoke Free Starts)***

**8 (A)** The video clip is worth remembering

- Strongly disagree
- Disagree
- Neither agree nor disagree
- Agree
- Strongly agree

**8 (B)** The video clip grabbed my attention

- Strongly disagree
- Disagree
- Neither agree nor disagree
- Agree
- Strongly agree

**8 (C)** The video clip is powerful

- Strongly disagree
- Disagree
- Neither agree nor disagree
- Agree
- Strongly agree

**8 (D)** The video clip is informative

- Strongly disagree
- Disagree
- Neither agree nor disagree
- Agree
- Strongly agree

**8 (E)** The video clip is meaningful

- Strongly disagree
- Disagree
- Neither agree nor disagree
- Agree
- Strongly agree

**8 (F)** The video clip is convincing

- Strongly disagree
- Disagree
- Neither agree nor disagree
- Agree
- Strongly agree

**8 (G)** I can relate to the video clip

- Strongly disagree
- Disagree
- Neither agree nor disagree
- Agree
- Strongly agree

**8 (H)** After watching the video clip, I feel more motivated to stop smoking

- Strongly disagree
- Disagree
- Neither agree nor disagree
- Agree
- Strongly agree

**8 (I)** After watching the video clip, I feel more likely to seek professional support to help me stop smoking

- Strongly disagree
- Disagree
- Neither agree nor disagree
- Agree
- Strongly agree

**8 (J)** After watching the video clip, I feel more positive about the mental health benefits of stopping smoking

- Strongly disagree
- Disagree
- Neither agree nor disagree
- Agree
- Strongly agree

**8 (K)** Do you have any other comments about this clip? Such as what you liked, or disliked (Maximum **100 Characters**)

________________________________________

9. Did you have a favourite video?

- Insert screenshot of video clip 1 (Concept 1)
- Insert screenshot of video clip 2 (Concept 2)
- Insert screenshot of video clip 3 (Smoke Free Starts)

**Demographics**

*The following questions are about you. We collect this information so we can see if our research includes a diverse range of people.*

10. What gender do you identify as?

- Male
- Female
- Prefer not to say
- Other __________________

**11.** What is your age?

- 18 –24
- 25 –34
- 35 – 44
- 45 – 54
- 55 - 64
- 65 +
- Prefer not to say

**12.** Choose one option that best describes your ethnic group or background

- Asian/Asian British
- Black/African/Caribbean/Black British
- Mixed/Multiple ethnic groups
- White
- Prefer not to say
- Other __________________

**13.** Where do you currently live?

- England
- Northern Ireland
- Scotland
- Wales
- Prefer not to say

**14.** What is the highest level of education that you have completed?

- GCSE/O-Level/CSE
- Vocational qualifications (=NVQ 1 + 2)
- A-level or equivalent (=NVQ 3)
- Bachelor degree or equivalent (=NVQ 4)
- Masters
- PhD or equivalent
- Other _____________
- No formal qualifications
- Still studying
- Don’t know
- Prefer not to say

**15.** What is your current employment status?

- Employed
- Self-employed
- Unemployed
- Unemployed due to disability/health reasons or on long term sick leave
- Retired
- Student
- Other __________
- Prefer not to say

**Attention Check**

The following question is to check your level of attention. This will ensure that the data are good quality.

**A2.** To check your level of attention, please tick **Sometimes**.

- Never
- Rarely
- Sometimes
- Often
- Very often
- Don’t know
- Prefer not to say

**Mental Health**

*The following questions ask a bit more about your mental health. We understand that this is a sensitive topic and any information you give is strictly confidential and will be used for research purposes only.*

**16.** During the past 30 days, about how often did you feel…

|  | All of the time | Most of the time | Some of the time | A little of the time | None of the time | Don’t know | Prefer not to say |
| --- | --- | --- | --- | --- | --- | --- | --- |
| …nervous |  |  |  |  |  |  |  |
| …hopeless |  |  |  |  |  |  |  |
| …restless or fidgety |  |  |  |  |  |  |  |
| …so depressed that nothing could cheer you up |  |  |  |  |  |  |  |
| …that everything was an effort |  |  |  |  |  |  |  |
| …worthless |  |  |  |  |  |  |  |

**17.** Which of the following, if any, has a doctor or healthcare professional ever told you that you had?

- Depression
- Anxiety
- Obsessive compulsive disorder (OCD)
- Schizophrenia
- Bipolar disorder (formally known as ‘Manic Depression’)
- Psychosis
- A personality disorder
- Panic disorder
- Problem gambling
- A phobia
- Alcohol misuse or dependence
- Drug use or dependence
- Post-traumatic stress disorder (PTSD)
- Attention deficit hyperactivity disorder (ADHD)
- An eating disorder (anorexia, bulimia, binge-eating disorder)
- Autism or autism spectrum disorder
- Other ___________
- None of these
- I am currently going through the diagnostic process
- Don’t know

Prefer not to say

The following information will be provided to everyone after Question 17

**Thank You for Taking the Time to Participate in this Survey.**

Please move on to Prolific by **clicking the proceed button/arrow.** You will automatically be re-directed to Prolific to register survey completion.

**Some Useful Information**

We all deal with diverse stressful situations, as such, mental health challenges can affect anyone and impact day-to-day life.

If you are currently experiencing a mental health crisis and your life may be at risk either:

- call 999 and ask for an ambulance
- go straight to A&E
- call your local crisis team

If you live in England, Scotland Wales or Norther Ireland and in need of urgent advice:

- call 111 or visit NHS 111 website: <https://111.nhs.uk/>
- contact your GP surgery and ask for an emergency appointment
- call an NHS urgent mental health helpline (if you live in England). You can search for a helpline number at the following website: <https://www.nhs.uk/service-search/mental-health/find-an-urgent-mental-health-helpline>

You can also contact the following organisations for mental health support. **Mind**

- Telephone: 0300 123 3393 (9am to 6pm, Monday to Friday (except for bank holidays))
- Email: [info@mind.org.uk](mailto:info@mind.org.uk)
- Website: [www.mind.org.uk](http://www.mind.org.uk/)

**Shout (text support service)**

- Text “SHOUT” to 85258 (free to text, 24 hours a day, 7 days a week)

**Samaritans**

- Telephone: 116 123 (free to call, 24 hours a day, 365 days a year)
- Email: [jo@samaritans.org](mailto:jo@samaritans.org)
- Website: [www.samaritans.org](http://www.samaritans.org/)

For information to enable you to decide on stopping smoking, please see the following guide

- [Stop smoking aids - NHS (www.nhs.uk)](https://www.nhs.uk/better-health/quit-smoking/stop-smoking-aids/)
- [Vaping to quit smoking - Better Health - NHS (www.nhs.uk)](https://www.nhs.uk/better-health/quit-smoking/vaping-to-quit-smoking/)
- [Find Your Local Stop Smoking Service (LSSS) - Better Health - NHS (www.nhs.uk)](https://www.nhs.uk/better-health/quit-smoking/find-your-local-stop-smoking-service/)
- [Free Personal Quit Plan - Quit Smoking - NHS (www.nhs.uk)](https://www.nhs.uk/better-health/quit-smoking/personal-quit-plan/)

**See Resources in Different Languages**

- Urdu - <https://www.smokefreestarts.co.uk/www/resource/smoking-and-mental-health-leaflet-urdu.pdf>
- Arabic - <https://www.smokefreestarts.co.uk/www/resource/smoking-and-mental-health-leaflet-arabic.pdf>
- Punjabi - <https://www.smokefreestarts.co.uk/www/resource/smoking-and-mental-health-leaflet-punjabi.pdf>
- Polish - <https://www.smokefreestarts.co.uk/www/resource/smoking-and-mental-health-leaflet-polish.pdf>
- Slovak - <https://www.smokefreestarts.co.uk/www/resource/smoking-and-mental-health-leaflet-slovak.pdf>
- Kurdish - <https://www.smokefreestarts.co.uk/www/resource/smoking-and-mental-health-leaflet-kurdish.pdf>
- Romanian - <https://www.smokefreestarts.co.uk/www/resource/smoking-and-mental-health-leaflet-romanian.pdf>
- Chinese - <https://www.smokefreestarts.co.uk/www/resource/smoking-and-mental-health-leaflet-chinese.pdf>
- Romani - <https://www.smokefreestarts.co.uk/www/resource/smoking-and-mental-health-leaflet-romani.pdf>
- English - <https://www.smokefreestarts.co.uk/www/resource/smoking-and-mental-health-leaflet-english.pdf>

**Re-direct to Prolific**

Automatically re-direct survey participants to Prolific using URL

<https://app.prolific.com/submissions/complete?cc=COYTV83S>
